# 3D Printed Microfluidic Features Using Dose Control in X, Y, and Z Dimensions

**DOI:** 10.3390/mi9070326

**Published:** 2018-06-28

**Authors:** Michael J. Beauchamp, Hua Gong, Adam T. Woolley, Gregory P. Nordin

**Affiliations:** 1 Department of Chemistry and Biochemistry, Brigham Young University, Provo, UT 84602, USA; mikejbeau@byu.edu; 2 Department of Electrical and Computer Engineering, Brigham Young University, Provo, UT 84602, USA; gonghuabupt@gmail.com

**Keywords:** 3D printing, microfluidics, particle traps, stereolithography

## Abstract

Interest has grown in recent years to leverage the possibilities offered by three-dimensional (3D) printing, such as rapid iterative changes; the ability to more fully use 3D device volume; and ease of fabrication, especially as it relates to the creation of complex microfluidic devices. A major shortcoming of most commercially available 3D printers is that their resolution is not sufficient to produce features that are truly microfluidic (<100 × 100 μm^2^). Here, we test a custom 3D printer for making ~30 μm scale positive and negative surface features, as well as positive and negative features within internal voids (i.e., microfluidic channels). We found that optical dosage control is essential for creating the smallest microfluidic features (~30 µm wide for ridges, ~20 µm wide for trenches), and that this resolution was achieved for a number of different exposure approaches. Additionally, we printed various microfluidic particle traps, showed capture of 25 µm diameter polymer beads, and iteratively improved the trap design. The rapid feedback allowed by 3D printing, as well as the ability to carefully control optical exposure conditions, should lead to new innovations in the types and sizes of devices that can be created for microfluidics.

## 1. Introduction

3D printing is a valuable technique for custom and rapid design change and optimization in fabrication of millifluidic devices [1]. Miniature device applications stand to benefit from the advantages offered from 3D printing, such as the ability to create and test devices with rapid feedback, allowing changes to be quickly tested. Device optimization based on empirical results could save time and money compared to traditional device fabrication techniques that involve conventional machining or micromachining.

A number of groups have recently sought to use 3D printing to produce fluidic devices for various applications. Devices for nitrite [2] or anemia [3] detection, measuring endocrine secretion [4], sorting bacteria [5], and cell culture [6,7], have all been shown to name a few. Although these are promising assays, a key issue from these works is the size of printed features. Most commercially available printers and resins are only able to achieve feature sizes down to 250 μm, with typical features around 500 μm, which are not suitable for many microfluidics applications. Additionally, these commercial printers lack flexibility in terms of resin development, individual layer custom exposure time control, or multiple exposures per layer. For 3D printing of fluidic devices with low surface roughness, stereolithography (SLA) printers are the best suited [8]. The material left in the channels after printing is a liquid and thus is much easier to clear than solid sacrificial materials formed with either polyjet or fused deposition modeling printers [9,10,11]. Reviews of 3D printing over the past several years have offered helpful insights regarding types of printers and their applications. Many different outlooks are given for future directions in 3D printing of fluidic devices, such as resin improvements, material removal techniques, throughput, and printer resolution [1,12,13,14,15,16]. Several groups have undertaken work to investigate and compare the resolution of various 3D printers in an effort to make fluidic devices [8,11,17,18,19]; however, much of this work is still well above 100 μm needed for most microfluidic applications.

Our group has developed an SLA 3D printer, as well as a custom resin formulated specifically for creating truly microfluidic structures with this printer [20], and we have made small (18 × 20 µm) microfluidic channels [21], as well as fluid control systems involving pumps and valves [22]. To make the smallest channels, an edge compensation technique was employed which overexposed the pixels at the channel edge to make it narrower [21]. However, we have not yet examined how this edge compensation approach affects features in the channels.

In this work, we investigate precise control over printing exposure areas and dosage conditions to create microscale substructures within microfluidic features. First, we look at positive and negative features on the exterior of prints to see what size features can be printed with various exposure times and with exposure edge compensation. Next, we evaluate positive and negative features in interior void areas to see the impact of exposure times and edge compensation. Finally, we create microfluidic particle traps to demonstrate how the ability to control specific dosing parameters allows improved function.

## 2. Materials and Methods 

### 2.1. Material Sources

Acetone, 2-propanol (IPA), and 25 μm polystyrene microspheres were purchased from Thermo-Fisher Scientific (Salt Lake City, UT, USA). Triethoxysilyl propylmethacrylate, hydroxypropyl cellulose (HPC) and polyethylene glycol diacrylate (PEGDA, 258 Da MW) were purchased from Sigma-Aldrich (Milwaukee, WI, USA). Toluene and glass microscope slides (3″ × 1″ × 1.2 mm) were purchased from Avantor (Center Valley, PA, USA). 2-Nitrodiphenylsulfide (NPS) was purchased from TCI America (Portland, OR, USA). Phenylbis (2,4,6-trimethylbenzoyl)phosphine oxide (Irgacure 819) was provided by BASF (Midland, MI, USA). All chemicals were used as received.

### 2.2. Glass Slide Preparation

Glass microscope slides were scored on one side using a laser cutter (Universal Laser Systems, Scottsdale, AZ, USA). The settings for cutting were 50% power, 10% speed, and 165 points per inch. The glass slides were then broken along the scored mark, washed with acetone and IPA, and dried with air. A fresh preparation of 10% triethoxysilyl propylmethacrylate in toluene was made. Glass slides were submerged in this silane solution in a shallow covered dish for a minimum of two hours, after which they were rinsed with IPA and dried with air. For longer term storage, slides were kept in a container under toluene.

### 2.3. Resin Preparation

The resin was prepared by mixing 2% NPS with 1% Irgacure 819 in 97% PEGDA. The resin was sonicated until all solid components dissolved and was stored in an amber bottle wrapped in aluminum foil to protect it from light. Details regarding resin formulation can be found in reference [21] for the choice of photoinitiator and absorber matched to the LED of the printer.

### 2.4. Device Designs

**Figure 1:**
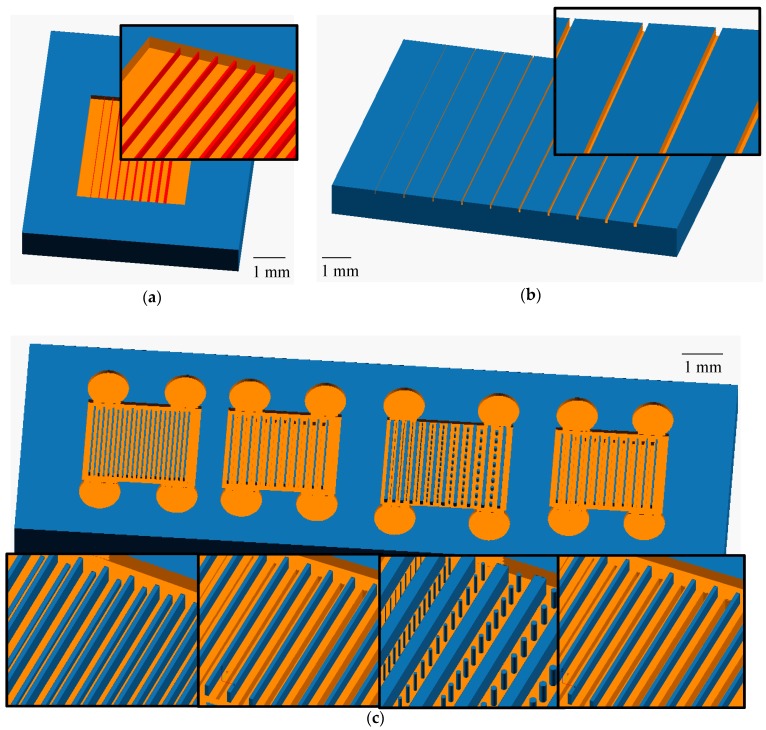

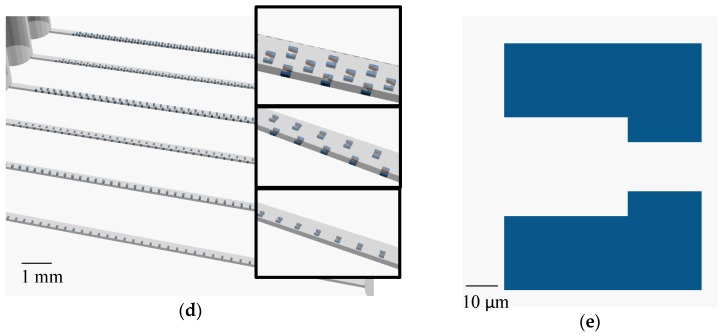
OpenSCAD designs of prints for exterior and interior resolution features. (**a**) Ridge device. The ridges are shown in red and there is a support box around the ridges. The ridges are 100 μm tall and have widths of 1–10 pixels (7.6–76 μm) from left to right. The cutout shows a zoom view; (**b**) Trench device with the trenches shown in orange. The trenches are 100 μm deep and have widths of 1–10 pixels (7.6–76 μm) from left to right. The cutout shows a zoom view; (**c**) Interior features device. The top layers of the device have been removed in the schematic to show the features. From left to right the regions are ridges, trenches with exposure compensation, pillars, and trenches without exposure compensation. Each set of features has a series of ceiling support ridges running the length of the feature area. Below each feature void area is a zoom view; (**d**) Bead trap device design showing 6 channels and traps within channels. Inserts show a zoom view of the three different trap layouts; (**e**) Schematic of bead trap. The large gap is designed to allow the beads to enter and the smaller gap allows fluid to pass through the trap.

Designs for 3D printed parts were made using open source OpenSCAD software (openscad.org). Schematics of the resolution prints can be seen in Figure 1. For the exterior ridges (Figure 1a) and trenches (Figure 1b), the features are 100 µm tall or deep, and the widths are from 1 to 10 pixels (7.6 to 76 µm), with a spacing between individual ridges or trenches of 100 pixels.

For the interior resolution features (Figure 1c), the height of the feature area is 100 µm. The internal ridges are all 5 pixels wide, 250 pixels long, and have heights from 1 to 10 layers (10 to 100 µm). For the trench sections, the trenches are all 100 µm deep and vary in width from 1 to 10 pixels. The pillars are designed with diameters ranging from 1 to 10 pixels, and all of the pillars in a given row are identical. In between each internal feature (or set of features with the pillars) is a support beam to help hold up the microchannel ceiling. These ceiling supports are all 5 pixels wide and go from the floor of the feature areas to the ceiling.

The trapping devices consist of 6 straight channels 30 pixels wide and 8 layers tall with fluidic reservoirs at both ends (Figure 1d). The traps consist of two L-shaped pieces facing each other that are 8 pixels long, 4 pixels wide, and spaced 2 pixels apart (Figure 1e). The traps are spaced 20 pixels apart down the length of the channel. Each print contains 6 different channels for testing a variety of trap layouts. Three different configurations of the traps were tested, one with traps only down the center of the channel, one with traps staggered along the edges, and one with traps staggered in the middle of the channel and along the sides (see Figure 1d, inserts).

### 2.5. 3D Printing Parameters

The 3D printer used for this work is the same as described in reference [21]. This printer operates with a nominally 385 nm light source and 7.6 µm pixel size in the image plane. The build layer height for all prints was 10 µm, the image plane irradiance was 21.2 mW·cm^−2^, and the exposure time was chosen to be either 500, 750, 1000, or 1500 ms to ensure thorough attachment of the print to the glass slide the first four layers were overexposed for 20, 10, 5, and 1 s, respectively. For prints in which the normal layer exposure time exceeded 1 s, only the first three layers were exposed in this manner. After printing, the remaining liquid resin in the print was flushed out three times with IPA using vacuum. Finally, the device was cured under an 11 mW 430 nm LED (ThorLabs, Newton, NJ, USA) for 10 min before use. This LED allows the photoinitiator to further cure the print at a wavelength at which the UV absorber (NPS) does not absorb the light.

### 2.6. Edge Compensation Technique

An edge compensation technique similar to the one in reference [21] was used where indicated for both interior and exterior trenches (negative features). This technique exposes the two pixels forming the edge of the trench for double the normal exposure time. The purpose of this technique is to cause a wider trench design to be narrower when printed. For example, a 3D printed trench that is designed to be 4 pixels wide without compensation has the same width as a 6 pixel wide design formed with compensation.

### 2.7. Measurement of Print Featuures

Exterior feature heights, depths, and widths were measured using a Zeta 20 optical profilometer (Zeta Instruments, San Jose, CA, USA). The width was measured as the full width at half height or depth. SEM imaging was done using an ESEM XL30 (FEI, ThermoFisher Scientific, Waltham, MA, USA). Samples were prepared by cutting them open with a razor blade and sputtering with 80:20 Au:Pd to allow the side profile to be observed. Images were processed using ImageJ (NIH) to measure the widths and heights of interior pillars, ridges, and trenches.

For trapping experiments, imaging was done using a Zeiss AXIO Observer A1 inverted microscope (Thornwood, NY, USA) using a 10× objective connected to a Photometrics coolSNAP HQ2 CCD camera (Tucson, AZ, USA). The exposure time for the CCD was 10 ms. The images were recorded and processed using ImageJ. Measurement of print features was performed three times (*n* = 3), and the standard deviation is given for each measurement.

For our printer and this resin, surface roughness has previously been characterized using optical profilometry with prints at various exposure times [22]. For all exposure times tested (600–1200 ms), the RMS surface roughness was less than 100 nm, typically 55–60 ± 15 nm. While some surfaces may appear to show pixelation (Figure 4 and Supplementary Materials) as they did in this previous work, it is expected that the surface roughness should still be the same.

### 2.8. Trapping Opperation

The bead solution for trapping was made by suspending the beads in deionized water at a concentration of 1 mg/mL with 0.5% HPC to prevent aggregation. 1.5 µL of bead solution was pipetted into the left reservoir as oriented in Figure 1d and drawn through the channel with vacuum over ~7 s, which resulted in a flow rate of 13 µL/min. This was repeated three times so a total of 4.5 µL of bead solution was pulled through the channel, after which CCD images were taken.

## 3. Results and Discussion

### 3.1. Exterior Features

#### 3.1.1. Ridges

**Figure 2:**
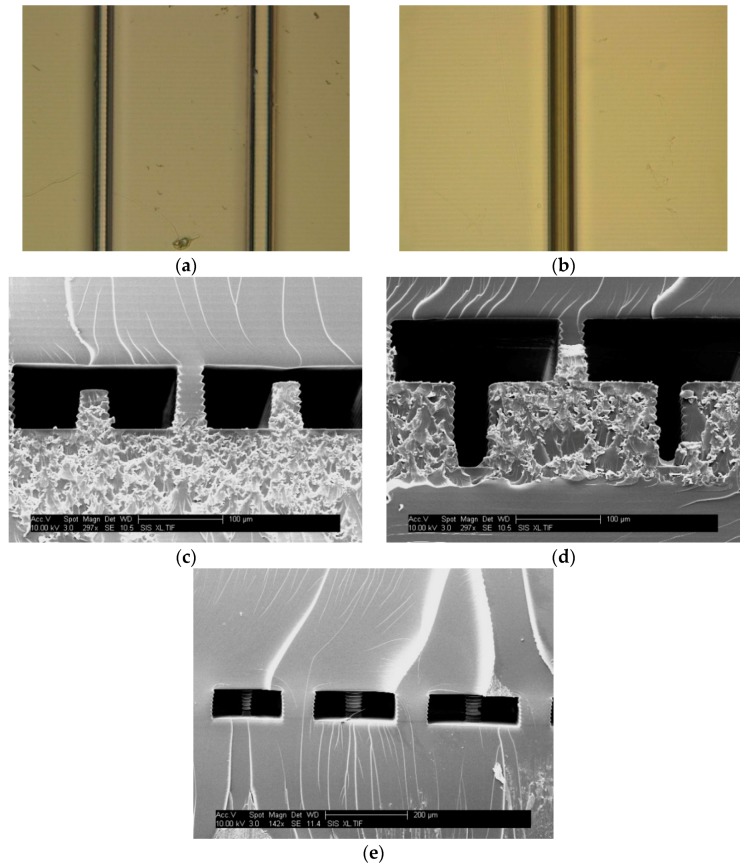
Images of 3D printed features. (**a**) Top view photograph of 1500 ms exposure ridges designed 3 and 4 pixels (23 and 30 µm) wide. The ridges measured 25 ± 1 and 29 ± 1 µm; (**b**) Top view photograph of a 500 ms exposure (without compensation) trench designed 4 pixels (30 µm) wide, which measures 21 ± 0.5 µm; (**c**) SEM images of 1000 ms exposure interior ridges designed 5 and 6 layers tall. The ridges measured 46 ± 1 and 55 ± 1 µm tall, respectively; a support pillar is in the middle of the image; (**d**) SEM image of interior trenches at 1000 ms exposure without compensation designed 5 and 6 pixels (38 and 46 µm) wide, which measured 22 ± 0.7 and 34 ± 2 µm wide; (**e**) SEM image of interior pillar structures at 1500 ms exposure designed to be 5–7 pixels (38–53 µm) in diameter.

**Figure 3:**
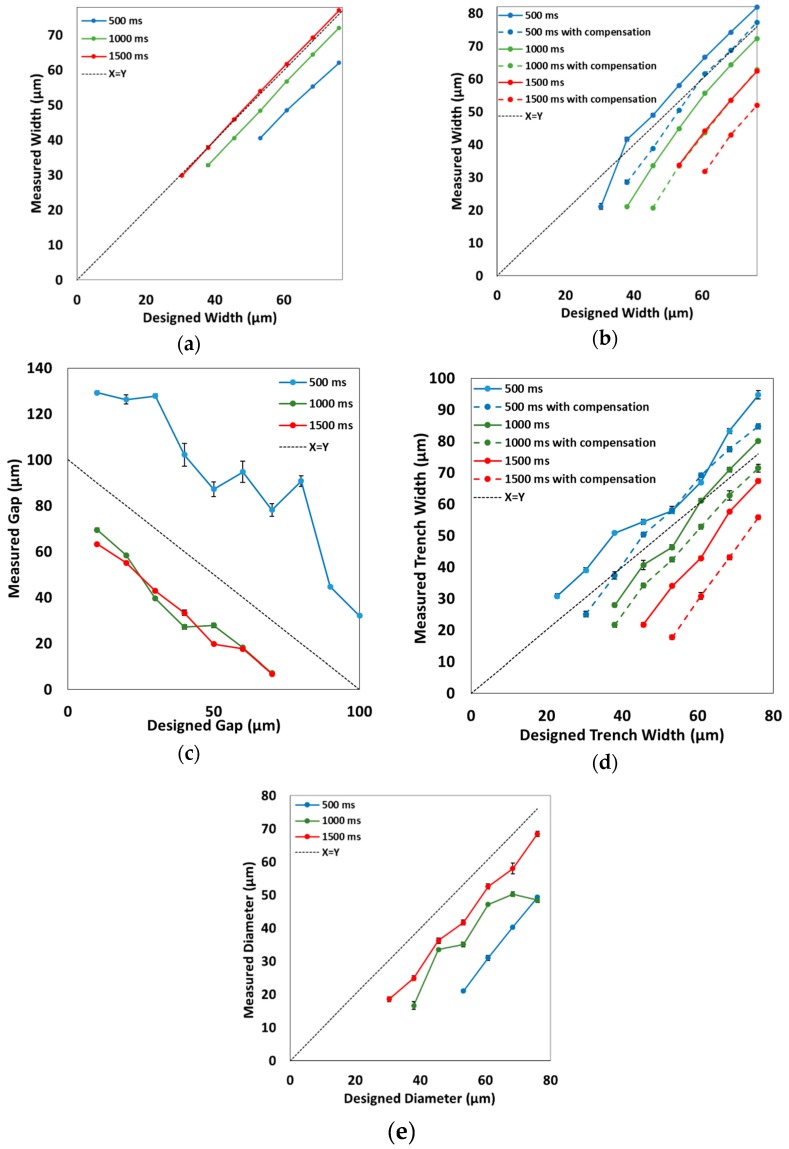
Designed feature width versus measured width for different exposure conditions. Only features that reached >90 µm in height (ridges) or depth (trenches) were included. For the pillars, only those that were attached floor to ceiling were included. Standard deviations are indicated for each included point (*n* = 3). (**a**) Exterior ridges; (**b**) Exterior trenches; (**c**) Interior ridges; (**d**) Interior trenches; (**e**) Interior pillars.

Initial testing focused on the exterior resolution features of our 3D printer by testing a set of ridges and trenches on the surface of prints. The purpose of these features is to evaluate how positive features turn out on the surface of print. For the surface ridges, the design shown in Figure 1a was created; the design was printed three times with exposure times of 500, 1000, or 1500 ms for each build layer. We found that the ends of the ridges became warped when they were not anchored, so a support box was placed around the ridges. A photograph though the microscope can be seen in Figure 2a, showing example ridges that are 3 and 4 pixels wide for 1500 ms exposure. The complete set of images can be found in the Supplementary Materials (Figures S1–S3). The heights and widths of these features were then measured with an optical profilometer. Figure 3a shows the measured ridge width plotted against the designed width for ridges that reached >90% of the full height. This shows that increasing light dosage from 500 ms (blue line in Figure 3a) to 1500 ms (red line in Figure 3a) allows smaller ridges to successfully reach full height and be closer to their designed width. Additionally, a ridge that is designed to a certain width will print smaller than expected if the exposure time is insufficient. The minimum width ridge that could be successfully printed was 30 ± 1 μm, which was with an exposure time of 1500 ms. These positive features benefit from increased light exposure, indicating the need to be able to give sufficient exposure to positive features.

#### 3.1.2. Trenches

For the exterior negative features, we created the design shown in Figure 1b. This design was printed with and without the exposure compensation pattern at 500, 1000, and 1500 ms for a total of six prints. An example microscope image showing a trench 4 pixels wide with 500 ms exposure can be seen in Figure 2b with the full data in Figures S4–S9. The heights and depths of these trenches were measured with optical profilometry. As seen in Figure 3b, measured width was plotted against designed width; only the features that achieved >90% of the designed feature depth were included. The first observation is that the compensation pattern caused the trenches to turn out narrower than the uncompensated devices due to the additional exposure at the trench edge. The second effect that can be seen is that increasing the layer exposure time for the uncompensated case results in narrower trenches, as expected. Finally we note that, to achieve a minimum trench width at full depth, there are three different possibilities: 500 ms exposure without compensation, 1000 ms exposure without compensation, or 1000 ms exposure with compensation. All three of these approaches produced a trench 100 µm deep and about 20 ± 0.5 µm wide; however, the designed widths were all different (4, 5, and 6 pixels, respectively). These results show that having precise control over both exposure and printing parameters allows for careful control of final feature sizes for exterior trenches.

### 3.2. Interior Features

For interior feature resolution, we undertook a similar study of positive and negative features in a confined space according to our design in Figure 1c. When prototyping this design, we found that a large void space without a support for the ceiling resulted in irregular top layers and erratic feature measurements. Thus, an alternating pattern of feature and ceiling support pieces exists in each of the interior feature areas. Each print contains two sets of trenches, either with or without exposure compensation, such that both could be tested in a single print. Additionally, Figure 2c–e shows characteristic SLA printing artifacts including uneven sides showing where each layer of the print sits on the next one, as well as a somewhat trapezoidal shape for the interior ridges and ceiling support pieces. Smaller layer thicknesses (finer z resolution) could help mitigate the layering, while the trapezoidal shape may be a result of material shrinkage.

#### 3.2.1. Ridges

We again used ridges as positive features to determine the types of structures that could be placed in an interior void in a 3D printed part. Five pixels width was chosen, because it formed reliably for surface ridges. An example SEM image of two ridges at 1000 ms exposure is shown in Figure 2c; the interior ridge 5 layers tall is shown on the left, with the ceiling support in the middle, and the ridge 6 layers tall is on the right. For interior ridges, we measured the gap between the top of the ridge and the ceiling of the void area, which gave information about interior z resolution. Gap distance (excluding any ridges that attached to the ceiling) is plotted for these interior ridges as shown in Figure 3c. The 500 ms exposure time resulted in a void region that was taller than the designed 100 µm, due to insufficient adhesion between layers, as can be observed in the Supplementary Materials (Figure S10). The ceiling support pieces appear to have broken off, resulting in voids taller than the designed size. For 1000 and 1500 ms, the void height is smaller than designed, likely due to exposure of the top layer of the chamber polymerizing significantly more than to 10 μm of resin, thereby making the first ceiling layer substantially thicker than designed, resulting in reduced overall chamber height and hence reduced gap size (Figures S11–S12). This is consistent with our previous work, in which we analyzed the layer exposure profile as a function of z [20]. The height of the void area was about 70–75 μm instead of 100 μm, and thus any ridges designed to be >7 layers tall were attached to the ceiling. For the ridges that were not attached, however, there was a linear relationship between the designed and measured gap distance. From this data, gaps between the feature and ceiling area as small as 7 ± 0.7 μm can be produced with either 1000 or 1500 ms exposures. As long as the print receives sufficient light exposure (>500 ms for this formulation), the gap height between features and ridges is independent of the exposure time for 1000 and 1500 ms exposures, as seen in Figure 3c.

#### 3.2.2. Trenches

For the interior trenches, a similar approach to the exterior trenches was used. The widths of printed trenches were measured with SEM imaging, and an example image is shown in Figure 2d for 1000 ms exposure (without compensation) of trenches that were designed to be 5 and 6 pixels (38 and 46 µm) wide. In this image the trench 6 pixels wide is on the left, and the trench 5 pixels wide is on the right, with the ceiling support pillar in the middle. The full set of interior trench images can been found in the Supplementary Materials Figures S13–S18. The measured width was compared to the designed width in Figure 3d, including only those trenches that reached >90% of full depth. The 500 ms trenches (both with and without compensation) turn out wider than the 1500 ms trenches, indicating that lower exposure times work better for forming wider trenches. Similar to the effect observed with the exterior trenches, the use of a compensation pattern leads to trenches smaller than they would have otherwise printed, as the compensated trenches are all narrower than the uncompensated ones. Similar to the exterior trenches, the smallest trenches that successfully formed were about 20 µm wide, though they were printed with different exposure times: 5 pixels wide for 1000 ms with compensation (21 ± 0.8 µm), 6 pixels wide for 1500 ms without compensation (21 ± 0.7 µm), and 7 pixels wide for 1500 ms with compensation (18 ± 0.7 µm). This again demonstrates the concept that there are several different ways to obtain a minimum feature size if the right exposure conditions are determined, especially when there is full control over the exposure properties.

#### 3.2.3. Pillars

A final type of resolution feature we investigated was cylindrical pillars in the interior of a void area. SEM images were taken of the pillars, and an example can be seen in Figure 2e showing 1500 ms exposure pillars that are 5–7 pixels wide (38–53 μm), with more data in Figures S19–S21 of the Supplementary Materials. Figure 3e shows a plot comparing the designed pillar diameter and the measured diameter, including only those pillars which were fully formed. From this graph, it can be seen that the pillars all printed narrower than their designed width and followed the trend that longer exposure times led to wider pillars. The smallest pillars that were successfully printed were about 14 ± 1 μm in diameter, printed with 1000 ms exposure for a 5 pixel designed width. The dependency of pillar diameter on light exposure is another example of how control over dosing parameters is essential to achieve desired feature dimensions during SLA 3D printing.

#### 3.2.4. Trapping Devices

**Figure 4:**
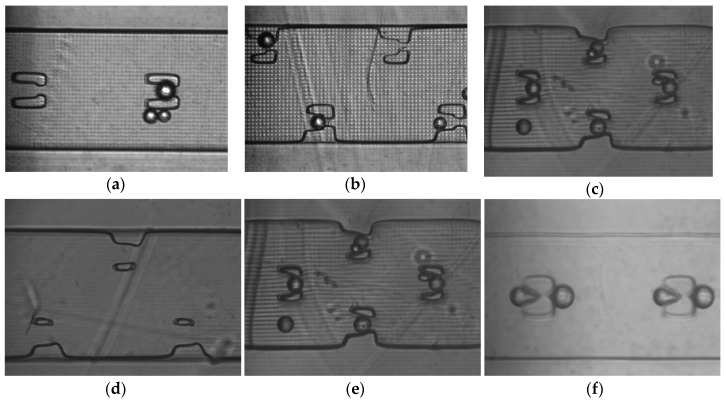
CCD images showing the effect of trap placement and different exposure times on trap shape. (**a**) Channel with traps exclusively in the center; (**b**) Channel with traps staggered along the sides; (**c**) Channel with traps staggered along the sides and in the middle of the channel; (**d**) Prints exposed 500 ms showing partially formed traps with no bead capture; (**e**) Prints exposed for 750 ms with beads trapped well; (**f**) Prints exposed at 1000 ms showing overexposed traps. Bubbles are stuck at the front and back of the traps hindering bead capture.

To demonstrate the utility of tight control of both positive and negative interior features we created trapping devices to catch particles as they flowed through a channel, as a first step toward trapping of cells. The beads selected were 25 μm in diameter, approximately the same size as the smallest trench that was successfully printed. Because our 3D printing designs can be easily edited to optimize configurations and print parameters, we attempted a number of different trap placements (see Figure 1d inserts) to determine the best trapping efficiency. Figure 4 shows images of channels after trapping experiments were carried out. We found that traps exclusively in the center of the channel (Figure 4a) did not trap as efficiently as traps along the sides (Figure 4b), which in turn were not as efficient as traps that alternated between the channel sides and center (Figure 4c). This was largely due to the flow of the beads around (instead of through) the traps. Additionally, we found that traps that were 3 layers tall did not trap as efficiently as traps that were the full height of the channel; when the traps are shorter many beads simply pass over the traps.

Finally, it is critical to have traps that have the correct dimensions. The degree of trap openness could be controlled through the exposure time used for the print. If the exposure time was too short (<600 ms), the traps would be partially formed, and the beads would pass through the trap without getting caught. Conversely, if the exposure time was too high (>1000 ms), the traps would end up overexposed, and there would be no flow through the traps, which would result in no beads being trapped and, often, small bubbles that stuck instead. The exposure time of ~750 ms was nearly ideal for forming traps that worked well for beads. Figure 4 shows this effect, with underexposed in (d), optimal exposure in (e), and overexposed in (f).

## 4. Conclusions

In this work, we have characterized the 3D printing of sub-100 μm external and internal positive and negative resolution features. We have shown the importance of controlling light dosage, as well as the benefits of multiple different exposure patterns within one layer of a print. Finally, we created a particle trapping device and leveraged the rapid iterative design capabilities of 3D printing to improve trap placement and efficiency.

These developments demonstrate the need for careful control of dosing parameters in making complex 3D printed microfluidic devices. More customization of printer control, resin development, and higher resolution projectors should lead to smaller, more intricate 3D printed microfluidic systems. Improved microfluidics of this type could provide even smaller traps, potentially allowing their use in cell capture or isolation experiments.

## Supplementary Materials

The following are available online at http://www.mdpi.com/2072-666X/9/7/326/s1: Figure S1: exterior features, ridges, 500 ms exposure; Figure S2: exterior features, ridges, 1000 ms exposure; Figure S3: exterior features, ridges, 1500 ms exposure; Figure S4: exterior features, trenches with edge compensation, 500 ms; Figure S5: exterior features, trenches with edge compensation, 1000 ms; Figure S6: exterior features, trenches with edge compensation, 1500 ms; Figure S7: exterior features, trenches without edge compensation, 500 ms; Figure S8: exterior features, trenches without edge compensation, 1000 ms; Figure S9: exterior features, trenches without edge compensation, 1500 ms; Figure S10: interior features, ridges, 500 ms; Figure S11: interior features, ridges, 1000 ms; Figure S12: interior features, ridges, 1500 ms; Figure S13: interior features, trenches with edge compensation, 500 ms; Figure S14: interior features, trenches with edge compensation, 1000 ms; Figure S15: interior features, trenches with edge compensation, 1500 ms; Figure S16: interior features, trenches without edge compensation, 500 ms; Figure S17: interior features, trenches without edge compensation, 1000 ms; Figure S18: interior features, trenches without edge compensation, 1500 ms; Figure S19: interior features, pillars, 500 ms; Figure S20: interior features, pillars, 1000 ms; Figure S21: interior features, pillars, 1500 ms.
