# Supplementary material for: 3D Printed Microfluidic Features Using Dose Control in X, Y, and Z Dimensions
Source: Micromachines (Basel). 2018 Jun 28;9(7):326. doi: 10.3390/mi9070326 (PMC6082287; doi:10.3390/mi9070326)
Supplement: Supplementary file 1 [file micromachines-09-00326-s001.pdf]

Supplementary Materials: 3D printed microfluidic features using dose control in X, Y, and Z dimensions

Michael J. Beauchamp, Hua Gong, Adam T. Woolley and Gregory P. Nordin

The supplementary information contains all the images used for measurements of interior and exterior features. The standard deviation is given for each measurement at n = 3. The tables are organized as follows:

1. Exterior Features S1–S9
    1.1. Ridges S1–S3
        500 ms exposure S1
        1000 ms exposure S2
        1500 ms exposure S3
    1.2. Trenches with edge compensation S4–S6
        500 ms exposure S4
        1000 ms exposure S5
        1500 ms exposure S6
    1.3. Trenches without edge compensation S7–S9
        500 ms exposure S7
        1000 ms exposure S8
        1500 ms exposure S9
2. Interior Features S10–S21
    2.1. Ridges S10-S12
        500 ms exposure S10
        1000 ms exposure S11
        1500 ms exposure S12
    2.2. Trenches with edge compensation S13–S15
        500 ms exposure S13
        1000 ms exposure S14
        1500 ms exposure S15
    2.3. Trenches without edge compensation S16–S18
        500 ms exposure S16
        1000 ms exposure S17
        1500 ms exposure S18
    2.4. Pillars S19–S21
        500 ms exposure S19
        1000 ms exposure S20
        1500 ms exposure S21

1. Exterior Features

1.1. Ridges

Designed Trench Width (μm)	76.0 and 68.4	60.8 and 53.2	45.6 and 38.0	30.4 and 22.8	15.2 and 7.6
Measured Trench Width (μm)	62.0 ±0.9 and 55.3 ±1.0	48.5 ± 2.2and 40.6 ± 0.9	30.7 ± 0.8 and 23.4 ±0.2	16.1 ±1.3 and 7.5 ±0.5	3.2 ±0.3 and -
Image	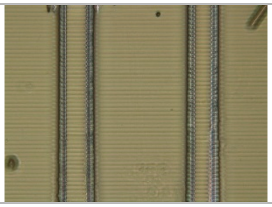	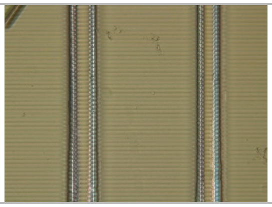	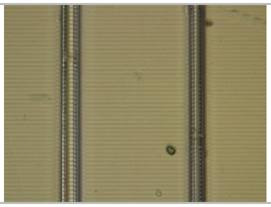	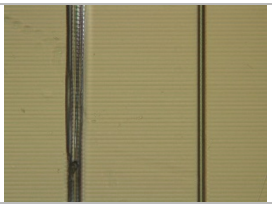	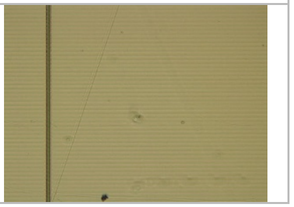

Figure S1. Exterior ridges exposed for 500 ms. Widths range from 10 to 1 pixels from left to right. The designed and measured widths are given as well as images of the ridges.

Designed Trench Width (μm)	76.0 and 68.4	60.8 and 53.2	45.6 and 38.0	30.4 and 22.8	15.2 and 7.6
Measured Trench Width (μm)	72.0 ± 0.3 and 64.4± 0.5	56.8 ± 0.3 and 48.4 ± 0.6	40.6 ±0.2 and 32.8 ± 0.9	24.9 ± 0.7 and 16.3 ± 1.5	7.2 ± 0.7 and 2.9 ± 0.4
Image	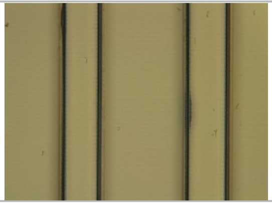	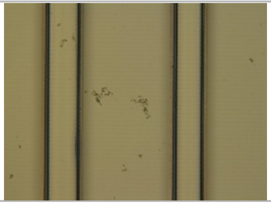	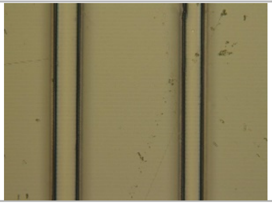	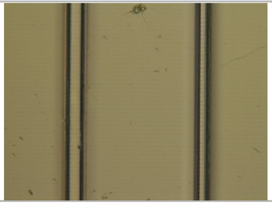	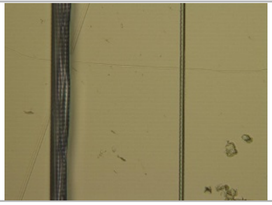

Figure S2. Exterior ridges exposed for 1000 ms. Widths range from 1 to 10 pixels from left to right. The designed and measured widths are given as well as images of the ridges.

Designed Trench Width (µm)	76.0 and 68.4	60.8 and 53.2	45.6 and 38.0	30.4 and 22.8	15.2 and 7.6
Measured Trench Width (µm)	77.1 ± 0.3 and 69.2 ± 0.4	61.7 ± 0.2 and 54.0 ± 0.3	45.9 ± 0.3 and 37.9 ± 0.3	29.8 ± 1.1 and 25.3 ± 1.3	15.1 ± 0.6 and 7.1 ± 0.8
Image	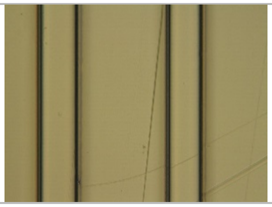	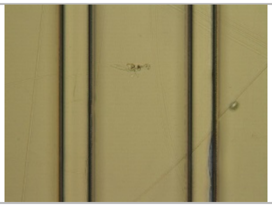	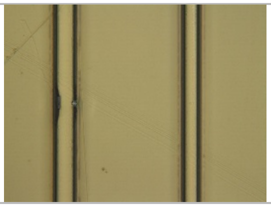	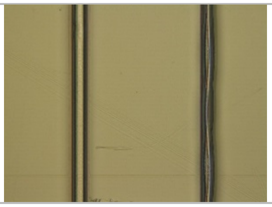	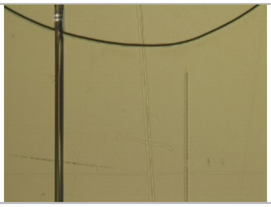

Figure S3. Exterior ridges exposed for 1500 ms. Widths range from 1 to 10 pixels from left to right. The designed and measured widths are given as well as images of the ridges.

1.2. *Trenches with edge compensation*

Designed Trench Width (µm)	76.0	68.4	60.8	53.2	45.6
Measured Trench Width (µm)	77.3 ± 0.6	68.7 ± 0.3	61.7 ± 0.4	50.5 ± 0.6	38.9 ± 0.4
Image	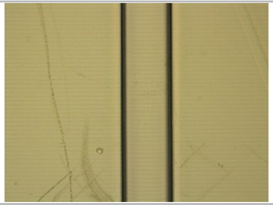	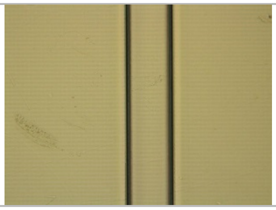	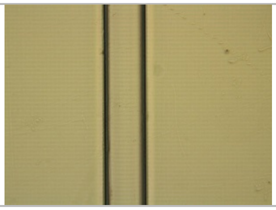	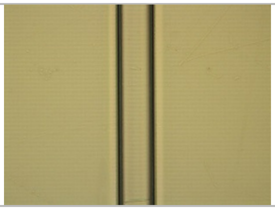	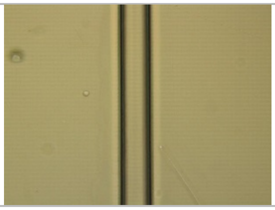
Designed Trench Width (µm)	38.0	30.4	22.8	15.2	7.6
Measured Trench Width (µm)	28.6 ± 0.7	5.5 ± 0.6	-	-	-
Image	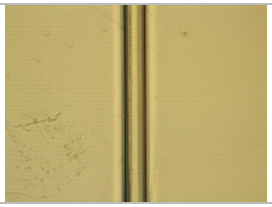	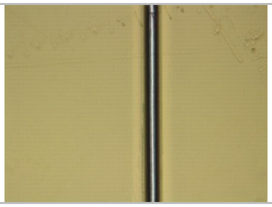	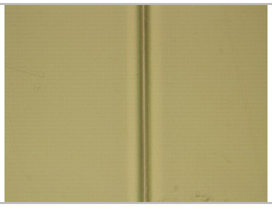	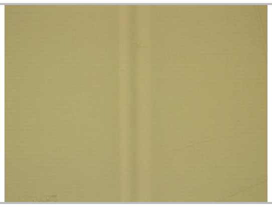	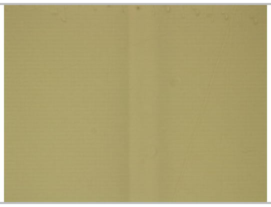

Figure S4. Exterior trenches with edge compensation exposed for 500 ms. Widths range from 10 to 1 pixels from left to right across two rows. The designed and measured widths are given as well as images of the trenches.

Designed Trench Width (μm)	76.0	68.4	60.8	53.2	45.6
Measured Trench Width (μm)	62.8 ± 0.2	53.5 ± 0.5	43.6 ± 0.6	33.5 ± 0.3	20.7 ± 0.4
Image	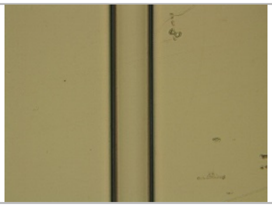	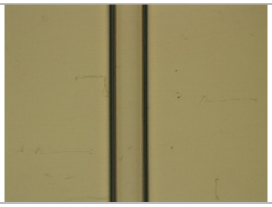	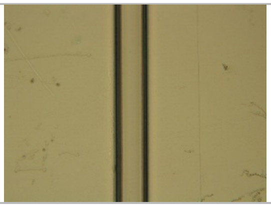	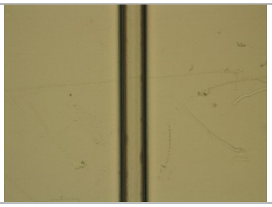	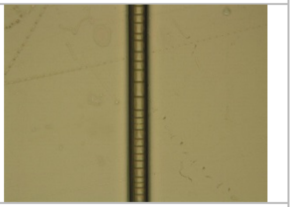
Designed Trench Width (μm)	38.0	30.4	22.8	15.2	7.6
Measured Trench Width (μm)	13.1 ± 1.9	-	-	-	-
Image	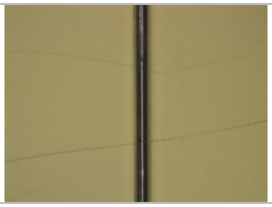	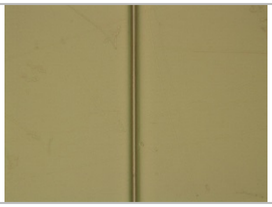	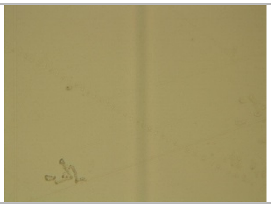	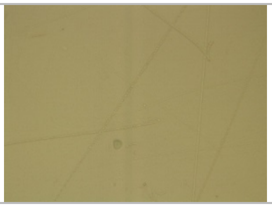	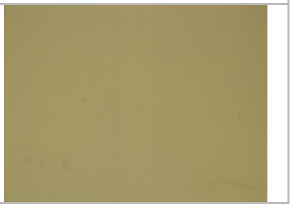

Figure S5. Exterior trenches with edge compensation exposed for 1000 ms. Widths range from 10 to 1 pixels from left to right across two rows. The designed and measured widths are given as well as images of the trenches.

Designed Trench Width (μm)	76.0	68.4	60.8	53.2	45.6
Measured Trench Width (μm)	52.0 ± 0.3	42.9 ± 0.5	31.8 ± 0.2	22.2 ± 0.5	11.4 ± 0.4
Image	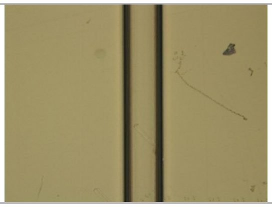	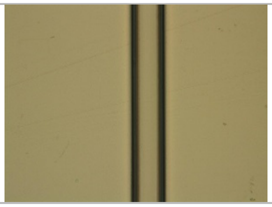	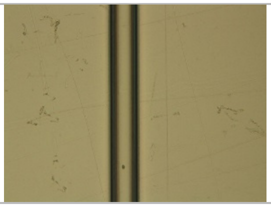	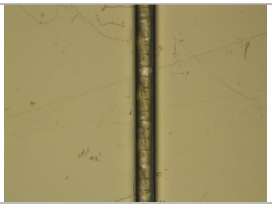	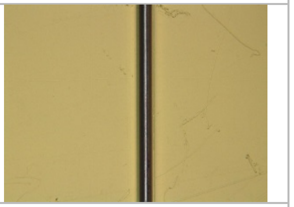
Designed Trench Width (μm)	38.0	30.4	22.8	15.2	7.6
Measured Trench Width (μm)	9.3 ± 0.7	-	-	-	-
Image	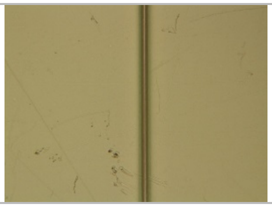	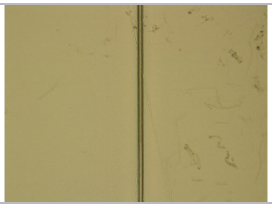	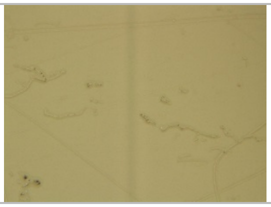	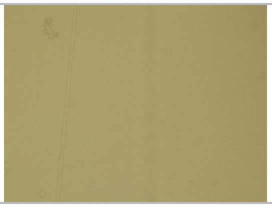	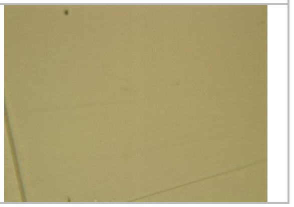

Figure S6. Exterior trenches with edge compensation exposed for 1500 ms. Widths range from 10 to 1 pixels from left to right across two rows. The designed and measured widths are given as well as images of the trenches.

1.3. Trenches without edge compensation

Designed Trench Width (μm)	76.0	68.4	60.8	53.2	45.6
Measured Trench Width (μm)	81.9 ± 0.5	74.3 ± 0.6	66.7 ± 0.9	58.1 ± 0.5	49.0 ± 1.2
Image	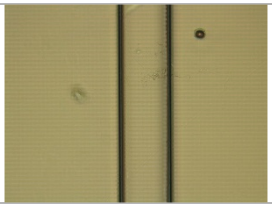	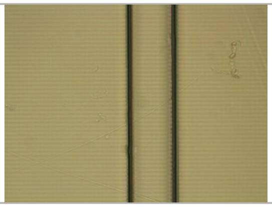	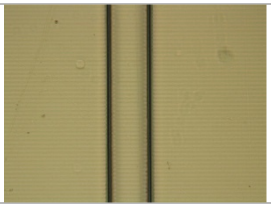	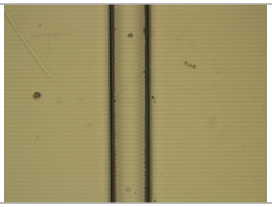	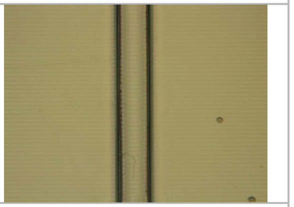
Designed Trench Width (μm)	38.0	30.4	22.8	15.2	7.6
Measured Trench Width (μm)	41.7 ± 0.4	21.1 ± 0.6	14.2 ± 0.6	10.1 ± 0.4	-
Image	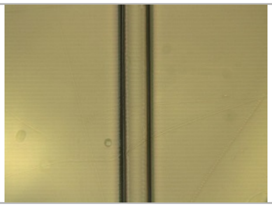	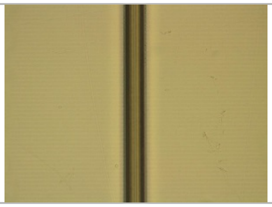	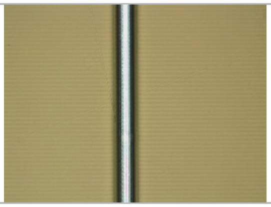	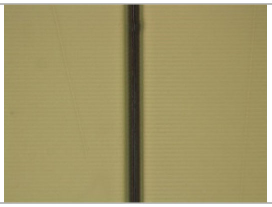	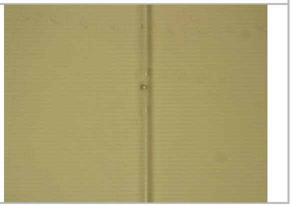

Figure S7. Exterior trenches without edge compensation exposed for 500 ms. Widths range from 10 to 1 pixels from left to right. The designed and measured widths are given as well as images of the trenches.

Designed Trench Width (μm)	76.0	68.4	60.8	53.2	45.6
Measured Trench Width (μm)	72.4 ± 0.3	64.3 ± 0.4	55.6 ± 0.2	44.9 ± 0.5	33.6 ± 0.4
Image	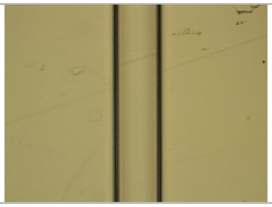	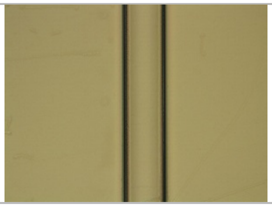	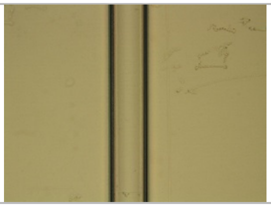	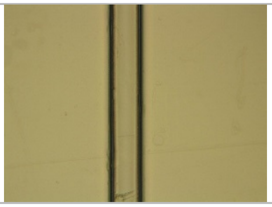	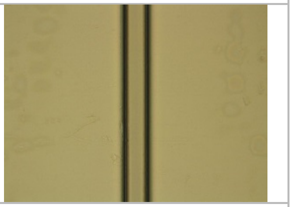
Designed Trench Width (μm)	38.0	30.4	22.8	15.2	7.6
Measured Trench Width (μm)	21.1 ± 0.3	12.9 ± 1.5	11.5 ± 0.9	-	-
Image	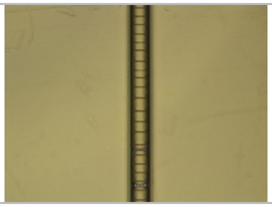	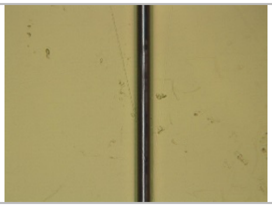	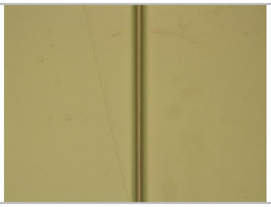	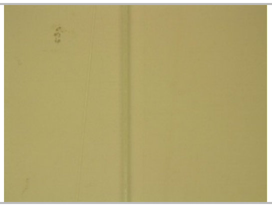	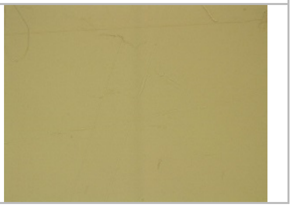

Figure S8. Exterior trenches without edge compensation exposed for 1000 ms. Widths range from 10 to 1 pixels from left to right across two rows. The designed and measured widths are given as well as images of the trenches.

Designed Trench Width (µm)	76.0	68.4	60.8	53.2	45.6
Measured Trench Width (µm)	62.4 ± 0.2	53.6 ± 0.1	44.2 ± 0.4	33.8 ± 0.2	24.7 ± 0.6
Image	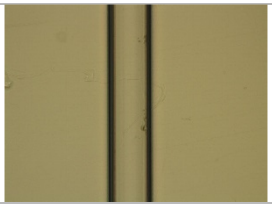	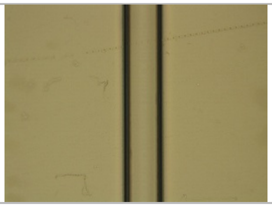	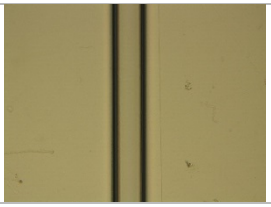	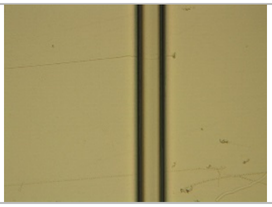	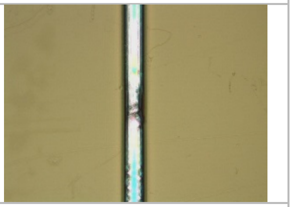
Designed Trench Width (µm)	38.0	30.4	22.8	15.2	7.6
Measured Trench Width (µm)	16.5 ± 0.3	8.6 ± 0.7	-	-	-
Image	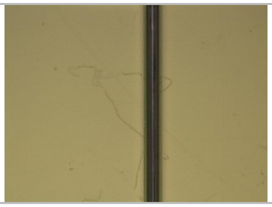	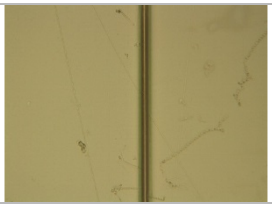	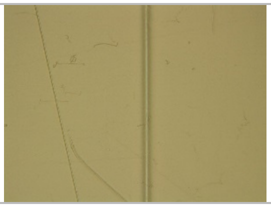	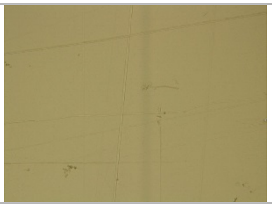	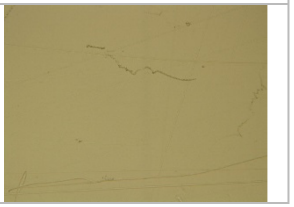

Figure S9. Exterior trenches without edge compensation exposed for 1500 ms. Widths range from 10 to 1 pixels from left to right across two rows. The designed and measured widths are given as well as images of the trenches.

2. Interior Features

2.1. Ridges

Designed Ridge Height (layers)	1 and 2	3 and 4	5 and 6	7 and 8	9 and 10
Measured Ridge Height (µm)	9.8 and 18.1	18.8 and 27.8	33.8 and 38.0	55.7 and 40.1	78.5 and 79.9
Measured Gap to Ceiling (µm)	129.3 ± 0.4 and 126.3 ± 0.8	127.8 ± 2.0 and 102.3 ± 0.9	87.2 ± 5.0 and 94.7 ± 3.1	78.2 ± 4.6 and 90.8 ± 2.8	44.8 ± 2.3 and 32.1 ± 0.4
Image	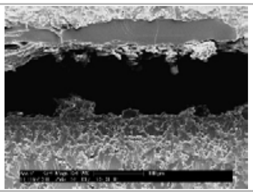	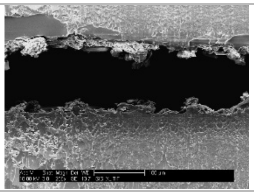	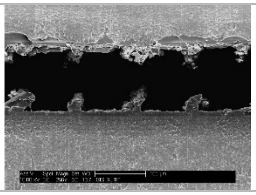	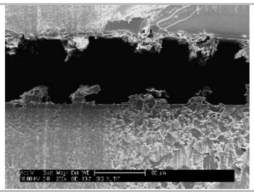	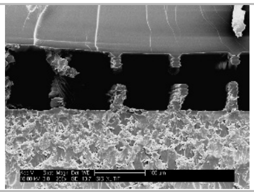

Figure S10. Interior ridges exposed for 500 ms. Heights range from 1 to 10 layers from left to right. The designed height, measured height, and height from top of the ridge to the top of the void feature (gap height), are given, as well as images of the ridges.

Designed Ridge Height (layers)	1 and 2	3 and 4	5 and 6	7 and 8	9 and 10
Measured Ridge Height (μm)	9.7 and 18.2	27.9 and 37.7	46.1 and 55.2	63.6 and 72.1	70.1 and 72.1
Measured Gap to Ceiling (μm)	69.5 ± 1.0 and 58.4 ± 0.8	39.6 ± 0.7 and 27.3 ± 0.7	27.9 ± 1.0 and 18.2 ± 1.0	7.1 ± 0.4 and -	- and -
Image	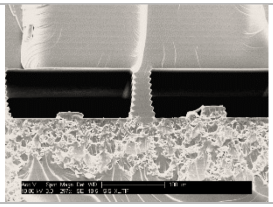	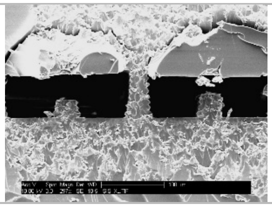	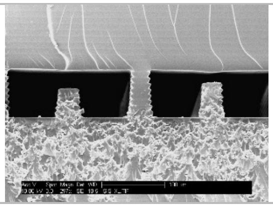	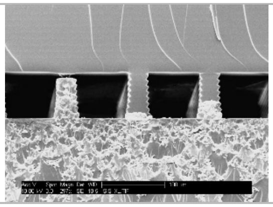	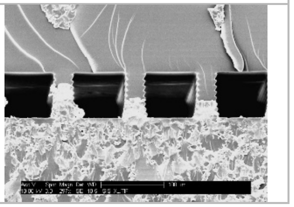

Figure S11. Interior ridges exposed for 1000 ms. Heights range from 1 to 10 layers from left to right. The designed height, measured height, and height from top of the ridge to the top of the void feature (gap height), are given, as well as images of the ridges.

Designed Ridge Height (layers)	10, 9, and 8	7 and 6	5 and 4	3 and 2	2 and 1
Measured Ridge Height (μm)	77.6, 74.8, and 73.5	69.4 and 62.6	48.3 and 38.8	29.3 and 19.1	19.1 and 9.5
Measured Gap to Ceiling (μm)	-, -, and -	6.8 ± 0.7 and 17.7 ± 0.4	19.7 ± 1.2 and 33.3 ± 0.7	42.9 ± 0.4 and 55.1 ± 0.7	55.1 ± 0.7 and 63.3 ± 0.7
Image	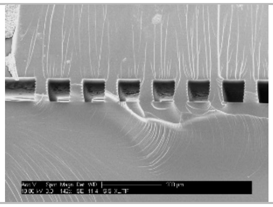	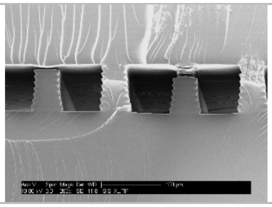	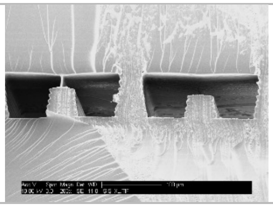	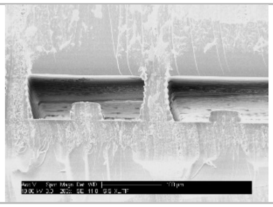	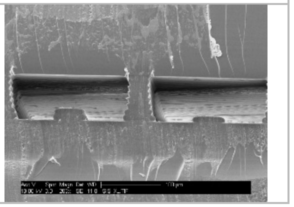

Figure S12. Interior ridges exposed for 1500 ms. Heights range from 10 to 1 layers from left to right. The designed height, measured height, and height from top of the ridge to the top of the void feature (gap height), are given, as well as images of the ridges.

2.2. Trenches with edge compensation

Designed Trench Width (µm)	7.6 and 15.2	22.8 and 30.4	38.0 and 45.6	53.2 and 60.8	68.4 and 76.0
Measured Trench Width (µm)	- and -	- and 25.1 ± 0.9	37.3 ± 1.2 and 50.4 ± 0.8	58.1 ± 1.2 and 69.2 ± 0.8	77.4 ± 0.8 and 84.7 ± 0.9
Image	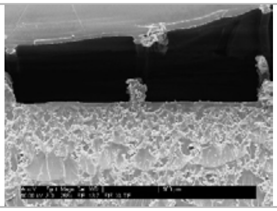	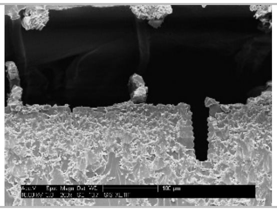	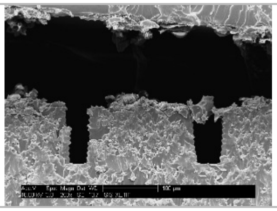	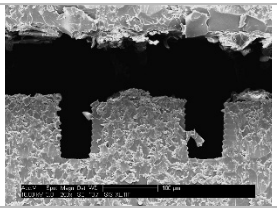	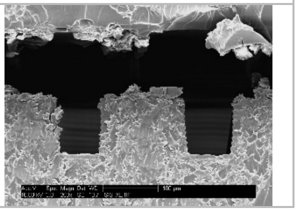

Figure S13. Interior trenches with edge compensation exposed for 500 ms. Widths range from 1 to 10 pixels from left to right. The designed and measured widths are given as well as images of the trenches.

Designed Trench Width (µm)	76.0, 68.4 and 60.8	53.2, 45.6, 38.0 and 30.4	22.8, 15.2, and 7.6
Measured Trench Width (µm)	71.4 ± 1.3, 62.8 ± 1.5, and 52.8 ± 0.8	42.4 ± 0.8, 34.2 ± 0.8, 21.6 ± 0.8, and -	-, -, and -
Image	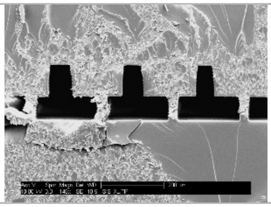	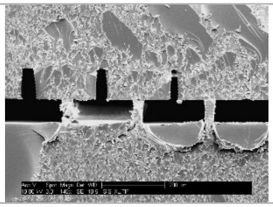	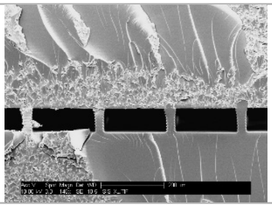

Figure S14. Interior trenches with edge compensation exposed for 1000 ms. Widths range from 10 to 1 pixels from left to right. The designed and measured widths are given as well as images of the trenches.

Designed Trench Width (μm)	7.6 and 15.2	22.8, 30.4, 38.0, and 45.6	53.2 and 60.8	68.4 and 76.0
Measured Trench Width (μm)	- and -	-, -, -, and -	17.7 ± 0.7 and 30.8 ± 1.0	43.1 ± 0.8 and 55.8 ± 0.7
Image	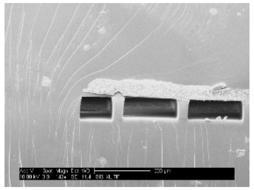	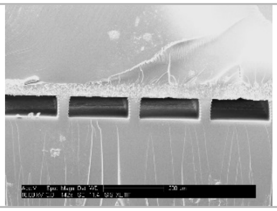	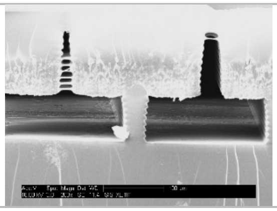	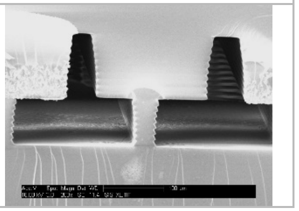

Figure S15. Interior trenches with edge compensation exposed for 1500 ms. Widths range from 1 to 10 pixels from left to right. The designed and measured widths are given as well as images of the trenches.

2.3. Trenches without edge compensation

Designed Trench Width (μm)	7.6 and 15.2	22.8 and 30.4	38.0 and 45.6	53.2 and 60.8	68.4 and 76.0
Measured Trench Width (μm)	- and -	30.8 ± 0.8 and 39.1 ± 0.8	50.9 ± 0.4 and 54.4 ± 0.9	57.9 ± 0.8 and 66.9 ± 0.8	83.2 ± 0.9 and 94.7 ± 1.3
Image	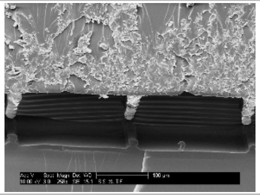	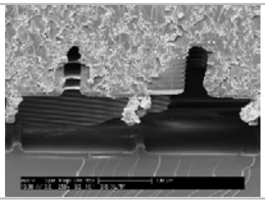	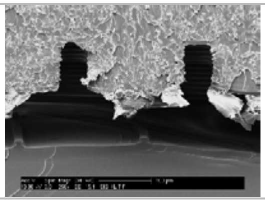	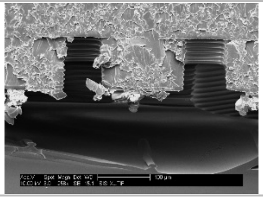	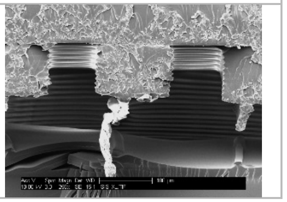

Figure S16. Interior trenches without edge compensation exposed for 500 ms. Widths range from 1 to 10 pixels from left to right. The designed and measured widths are given as well as images of the trenches.

Designed Trench Width (µm)	76.0 and 68.4	60.8 and 53.2	45.6 and 38.0	30.4 and 22.8	15.2 and 7.6
Measured Trench Width (µm)	80.1 ± 0.4 and 71.0 ± 0.7	61.0 ± 0.7 and 46.3 ± 0.8	40.7 ± 1.5 and 27.9 ± 0.7	- and -	- and -
Image	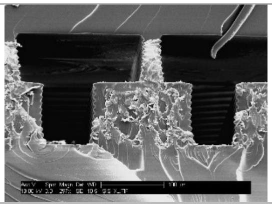	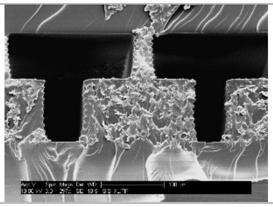	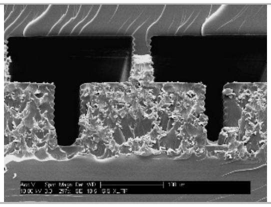	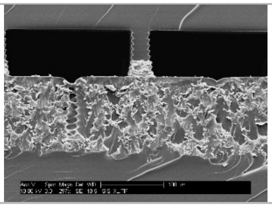	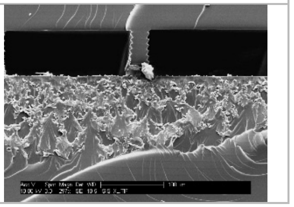

Figure S17. Interior trenches without edge compensation exposed for 1000 ms. Widths range from 10 to 1 pixels from left to right. The designed and measured widths are given as well as images of the trenches.

Designed Trench Width (µm)	7.6, 15.2, 22.8, and 30.4	38.0 and 45.6	53.2 and 60.8	68.4 and 76.0
Measured Trench Width (µm)	-, -, -, and -	- and 21.8 ± 0.7	34.0 ± 0.4 and 42.8 ± 0.7	57.6 ± 0.4 and 67.4 ± 0.7
Image	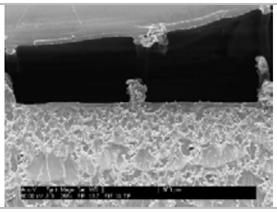	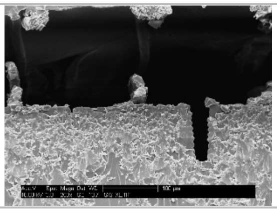	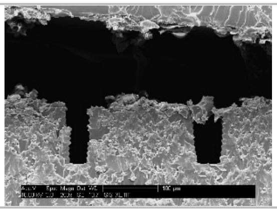	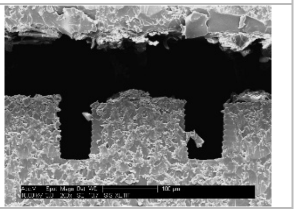

Figure S18. Interior trenches without edge compensation exposed for 1500 ms. Widths range from 1 to 10 pixels from left to right. The designed and measured widths are given as well as images of the trenches.

2.4. Pillars

Designed Pillar Diameter (µm)	7.6, 15.2, and 22.8	30.4 and 38.0	45.6 and 53.2	60.8 and 68.4	76.0
Measured Pillar Diameter (µm)	-, -, and -	- and -	19.3 ± 0.4 and 21.1 ± 0.8	31.1 ± 0.4 and 40.4 ± 0.4	49.4 ± 0.4
Image	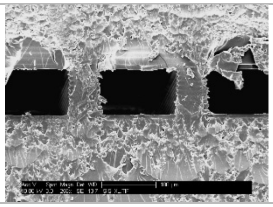	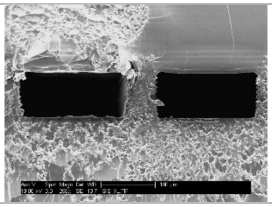	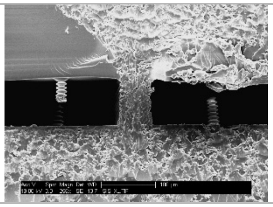	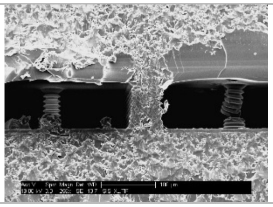	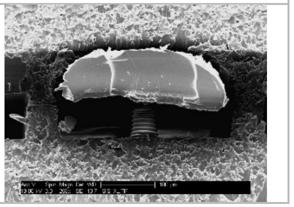

Figure S19-Interior pillars exposed for 500 ms. Widths range from 1 to 10 pixels from left to right. The designed and measured diameters are given as well as images of the pillars.

Designed Pillar Diameter (µm)	76.0 and 68.4	60.8 and 53.2	45.6 and 38.0	30.4 and 22.8	15.2 and 7.6
Measured Pillar Diameter (µm)	48.5 ± 0.8 and 50.2 ± 0.8	47.2 ± 0.8 and 35.1 ± 0.1	33.6 ± 0.8 and 16.7 ± 0.4	13.6 ± 1.1 and -	- and -
Image	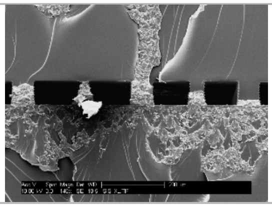	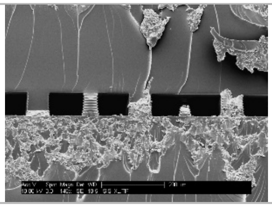	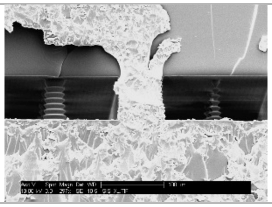	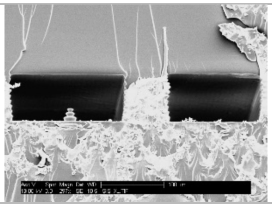	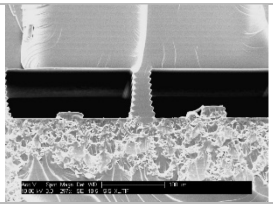

Figure S20. Interior pillars exposed for 1000 ms. Widths range from 10 to 1 pixels from left to right. The designed and measured diameters are given as well as images of the pillars.

Designed Pillar Diameter (µm)	7.6, 15.2, 22.8 and 30.4	38.0, 45.6, and 53.2	60.8, 68.4 and 76.0
Measured Pillar Diameter (µm)	-, 12.2 ± 2.4, -, and 18.6 ± 0.8	24.9 ± 0.8, 36.3 ± 0.8, and 41.7 ± 0.8	52.6 ± 0.8, 58.0 ± 1.6, and 68.5 ± 0.8
Image	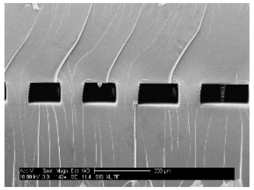	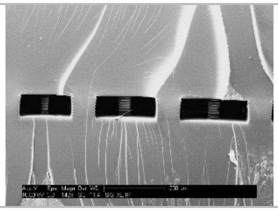	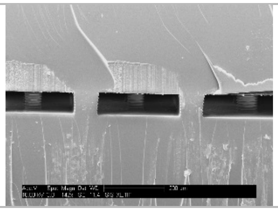

Figure S21. Interior pillars exposed for 1500 ms. Widths range from 1 to 10 pixels from left to right. The designed and measured diameters are given as well as images of the pillars.
